# Healthcare professionals' perceptions of psychosocial support for family members in palliative care inpatient units—A qualitative descriptive study

**DOI:** 10.1002/nop2.1548

**Published:** 2022-12-20

**Authors:** Anu Soikkeli‐Jalonen, Kaisa Mishina, Heli Virtanen, Andreas Charalambous, Elina Haavisto

**Affiliations:** ^1^ Department of Health Sciences, Faculty of Social Sciences Tampere University Tampere Finland; ^2^ Department of Nursing Science University of Turku Turku Finland; ^3^ Department of Child Psychiatry University of Turku Turku Finland; ^4^ INVEST Research Flagship Centre University of Turku Turku Finland; ^5^ Cyprus University of Technology Limassol Cyprus; ^6^ Satakunta Central Hospital Pori Finland

**Keywords:** families, hospital, interprofessional healthcare, palliative care, qualitative

## Abstract

**Aim:**

This study aimed to describe the psychosocial support healthcare professionals in specialist palliative inpatient units provide to family members of palliative care patients.

**Design:**

A qualitative descriptive design.

**Method:**

The data were collected with focus group interviews and analysed with inductive content analysis.

**Results:**

Altogether, 48 healthcare professionals, including physicians, registered nurses and practical nurses, participated in the study. Information sharing was recognised as an essential element of support. Methods to improve support of family members included an opportunity to allocate recourses to the families, systematic support and strengthening healthcare professionals' competence in family care.

The healthcare professionals describe their perceptions of psychosocial support for family members primarily through patient care and the patient's situation rather than family needs. Direct support for the family members is realised principally by information sharing and conversations. Healthcare professionals express their opportunities to implement support focusing on family members' needs restricted by reason of organisational resources.

The information can be used when developing and improving family care in palliative care context to recognise the most relevant needs from healthcare professionals' perspective and also when implementing healthcare professionals' education and training.

## 1 INTRODUCTION

Palliative care influences millions of people annually, including patients' family members (Connor, 2020). Family support is an indispensable part of palliative care provision, which intends to consider and care for the family members' needs (Steele & Davies, 2015). Support for family members is often implemented primarily through psychosocial support, which typically aims to not only answer the family members' psychological, emotional, informational, social, cultural and spiritual needs, but it can also involve practical support (Macleod, 2008). Healthcare professionals play an essential role in supporting family members of palliative care patients (LaValley, 2018). That is particularly evident in hospital care, where family members value the professional and safe care of the patient; they expect support from the healthcare professionals, including empathy, compassion, involvement in the care and privacy about their personal space (Virdun et al., 2017). However, families are not always sufficiently considered when implementing palliative care services, and their needs are not recognised enough (Areia et al., 2019). Family members have numerous unmet needs concerning their own coping (Ullrich et al., 2021; Wang et al., 2018), and their need for support can be even higher than patients themselves, especially in inpatient care (Oechsle et al., 2019).

## 2 BACKGROUND

Family members need support in palliative care, especially during the patients' hospital periods, and the hospital environment even increases their support needs (Lee & Cha, 2017). Although family members' needs are often related to their coping (Ullrich et al., 2021) and psychosocial needs, the need for various services has also been described (Wang et al., 2018). Additionally, family members require support from healthcare professionals to attend to patient care and be present in the care unit (Saarinen et al., 2021; Soikkeli‐Jalonen et al., 2021). The chance to participate in care activities is important to family members, and it is an essential element of family members' experience of support (Virdun et al., 2017). Furthermore, appropriate information sharing about a patient's diagnosis and impending death has been found to benefit family members (Røen et al., 2019). Moreover, bereavement support organised after a patient's death is found to be beneficial in helping with family members' grief management and ability to cope psychologically (Kustanti et al., 2021).

Supporting family members living in a distressing situation is complex, and improving family members' well‐being can be challenging (Saarinen et al., 2021; Soikkeli‐Jalonen et al., 2021). In addition, the physical care environment, culture and attitudes towards the family members in the care unit may enhance or hinder support (Partanen et al., 2018). When the care environment is experienced as positive, it can enhance family members' feeling of being supported by the healthcare professionals (Saarinen et al., 2021; Soikkeli‐Jalonen et al. 2021; Virdun et al., 2017). However, it has been observed that the healthcare system is designed mainly for the patients, with family members' needs coming second (Røen et al., 2019), and family members often remain unsupported by healthcare professionals, especially with regard to their psychological stress (Salifu et al., 2021). Supporting family members has not been the main focus of palliative care training (Teixeira et al., 2019), and healthcare professionals do not necessarily have sufficient competence to support family members in a palliative care context; they generally have more knowledge about the physical aspects of the care process (Røen et al., 2019). It can lead to problems in recognising family members' needs and implementing support (Teixeira et al., 2019).

There are few studies on supporting family members in inpatient palliative care (Saarinen et al., 2021; Soikkeli‐Jalonen et al., 2021). The role of families in palliative inpatient care needs further study, as families can easily feel like outsiders in a foreign hospital environment and need support from healthcare professionals to achieve a sense of comfort and ease (Partanen et al., 2018).

Not many studies disclose how support is provided in a palliative care setting from the viewpoint of healthcare professionals or their opportunities for implementing support in daily care, even though their role in providing support for family members is essential (Røen et al., 2019). Even though family members requirements and dissatisfaction about the support during patients' palliative care is stated in studies (Ullrich et al., 2021; Wang et al., 2018), the current research does not reveal healthcare professionals' perspective of their position in family support delivery or tell what the professionals experience of their real possibilities to support families in practice. There is a lack of studies displaying what do the healthcare professionals perceive as essential for palliative care family support and what are their experiences of employing the support in practice. The healthcare professional's perspective is essential, as they are the key providers of the support (Røen et al., 2019; Teixeira et al., 2019), and their competence and attitudes are the main promoting or inhibiting factors when developing and improving the palliative care support practices (Soikkeli‐Jalonen et al., 2020).

## 3 AIM

This study aims to describe the healthcare professionals' perceptions of how psychosocial support is provided to family members in palliative inpatient units and how this support can be improved.

## 4 METHOD

### 4.1 Study design and setting

A qualitative descriptive design was applied. The study was conducted in six specialist palliative care units in five hospitals which are part of two large hospital districts in (Saarto & Finne‐Soveri, 2019). Specialist palliative care can be described as holistic care for patients and families, and the healthcare professionals working in specialist units are expected to have expertise (Forbat et al., 2020). In Finland, specialist palliative care is centralised in units whose primary task is arranging palliative care, including end‐of‐life care. According to official guidelines, the care personnel are recommended to have specific palliative‐care training (Saarto & Finne‐Soveri, 2019). The consolidated criteria for reporting qualitative studies (COREQ): 32‐item checklist was used to ensure detailed and comprehensive reporting during the analysis and reporting process (Tong et al., 2007).

### 4.2 Participants

Purposive sampling was used to identify informants who had the most appropriate knowledge and understanding of the research subject (Krueger, 2015). Healthcare professionals responsible for planning and performing the daily care of palliative care patients and their families were invited. The inclusion criteria for healthcare professionals were that they must be either physicians, Registered Nurses or practical nurses who were permanently employed in a specialist palliative care inpatient unit. All meeting the inclusion criteria and willing to participate were included, and the number of participants were from eight to 15 from each participating hospital.

### 4.3 Data collection

Data were collected from May to September 2019 by three female interviewers (a Bachelor in Nursing Science, PhD candidate, and PhD), all Registered Nurses with palliative care experience but no professional relationship with the participants. Before the interviews, the participants were informed about the interviewers' role and position in the research project. Recruitment was based on voluntary participation, and information about the study was revealed to the participating organizations before recruitment by the principal investigator. The number of participants in the different occupational groups was proportional to the number of professionals working in the units. The interviews were conducted in a quiet room in a hospital environment, where participation was easy to implement about the work requirements of the participants.

The interview questions were following the study questions based on previous literature about the family members psychosocial support (Aoun et al., 2017; Papastavrou et al., 2012; Wang et al., 2018), but were open‐ended and broad to enable healthcare professionals to express their perceptions accurately. The interviews included questions like: ‘How are families supported?’, ‘What informational/emotional/other support do you provide for the families?’ and ‘How would you improve family support?’.

The data were collected through focus groups of two‐ to six‐person interviews to provoke a lively debate with various views on the subject represented (Krueger, 2015). Individual interviews were arranged if participation in the focus group could not be arranged, and these interviews were done primarily with doctors, as not many of them work in the same unit. The duration of the interviews ranged between 36 min and 2 h 50 min (Table 1), and the transcribed material contained 248 A4 pages with single‐line spacing. In addition, demographic information about participants was collected. The data saturation reached code saturation, and the data were collected until a good viewpoint of all professional groups had been gathered to get a deep view of the categories' content to describe the phenomenon (Hennink et al., 2017). All interviews were recorded via audio.

**TABLE 1:** Demographic information

Professional group	Interview type	Professionals involved	Duration (mean)	Work experience in healthcare (mean)	Work experience in palliative care (mean)	Age (mean)
All		*n* = 48	57 min	15.1 years	5 years	46 years
Registered Nurses		*n* = 30	1 h 38 min	15 years	5.2 years	42.3 years
	FGN1	6				
	FGN2	4				
	FGN3	3				
	FGN4	3				
	FGN5	4				
	FGN6	5				
	FGN7	3				
Practical nurses		*n = 13*	45 min	12 years	4.4 years	40.6 years
	FGPN1	5				
	FGPN2	4				
	FGPN3	4				
Physicians		*n = 5*	54 min	23.4 years	6.3 years	51.2 years
	FGP1	2				
	FGP2	1				
	FGP3	1				

### 4.4 Data analysis

Inductive content analysis was used to create a conceptual structure to describe healthcare professionals' perceptions (Elo & Kyngäs, 2008). Inductive content analysis was selected, because the previous research about the topic was limited and there was need to analyse the data to find new insights to the phenomenon. Furthermore, inductive concept analysis allows data categorisation, which was suitable when processing the unstructured qualitative data (Elo & Kyngäs, 2008).

In the first stage, recorded interviews were transcribed verbatim, and the unit of analysis was selected. In this study, the unit of analysis was a sentence or expression longer than one word that described a healthcare professional's perception. As the aim was to describe healthcare professionals' perceptions, only the manifest data were analysed to ensure the reliability. Second, the entire document was read multiple times to form a general understanding of the data and to conduct open coding by adding highlighting, notes and headings to the text to get preconception about the possible categories. Third, after the open coding, the units of analysis were tabulated, and simplified expressions for them were then established, coded and listed under categories by a researcher. After the data were tabulated and coded, the inductive content analysis was continued by a research group of four researchers to interpret the data and describe the phenomenon. Subcategories were formed by combining related codes of the expressions and interpreting what content belong into same categories and the categories were named with an expression that described it in the most representative way. The analysis generated categories which were united to subcategories with similar content. Finally, subcategories with related content were combined into main categories (Table 2). All data from different participating professional groups were analysed together as a single dataset.

**TABLE 2:** Healthcare professionals' perceptions of psychosocial support for family members

Subcategory	Category	Main category
Care meetings	Keeping the family member informed of the care	Informational support for family members
General information sharing		
Helping a family member to understand a patient's situation	Requirements for information sharing	
Providing direct information		
Respecting a patient's will to deny information sharing with family		
Taking care of family members' well‐being	Supporting family needs	Comprehensive support for family members
Organising additional support for a family member		
Creating confidence that a patient receives good care	Facilitating and mediating family members' and patients' togetherness	
Enabling the presence of family members		
Encouraging family members' participation		
Healthcare professionals' compassionate presence with family members	Supportive encounters	
Conversations with family members		
A better physical environment for families	Allocating resources to the family members' support	Methods to improve support for family members
More time for encounters with the families		
Better consideration of family support	Coordinating formal and informal support for family members	
Development of bereavement support		
Informal events for families		
Better communication skills	Developing professional competence	
Education about family care		

### 4.5 Ethics

The study was approved by the Ethics Committee of (REDACTED) (approval number REDACTED). Research permissions were obtained from each hospital accordingly. Ethical standards about the participants' autonomy, privacy, self‐determination and anonymity were followed (World Medical Association, 2013). Written informed consent was obtained previously from all participants before the study. The datasets produced and analysed are not publicly available to protect participants' privacy.

## 5 RESULTS

Altogether, 48 healthcare professionals participated in the study. Additional demographic information is presented in Table 1.

### 5.1 Healthcare professionals' perceptions of psychosocial support

The healthcare professionals' perceptions of psychosocial support for family members produced three main categories: *informational support for family members*, *comprehensive support for family members* and *methods to improve support for family members*. The categorisation is shown in Table 2.

#### 5.1.1 Informational support to family members

The first main category contained two categories: *keeping the family member informed of the care* and *requirements for information sharing*.

*Keeping the family member informed of the care* included the subcategories of *care meetings* and *general information sharing*. Informing family members was implemented by allowing family members to be present at *care meetings* in which a patient's situation was discussed and planned in a multi‐professional group of healthcare professionals and patients.

*FGN3*: ‘We arrange care meetings together with family members, and a doctor, a nurse, relatives, and the patient are present’.

The physician's role in information sharing during the care meetings was emphasised, and new information about a patient's condition or prognosis was seen as the physician's responsibility to share. It was also mentioned that physicians spent considerable time talking with family members.

*FGPN1*: ‘If there are any questions regarding the disease or prognosis … then it is a doctor [who shares the information]’.

*FGP2*: ‘The best way [to share information with a family member] is to involve the family members in the appointment’.

*General information sharing* through verbal interaction, providing information leaflets, calling or contacting, and keeping family members informed of updates were described as standard supportive procedures. In addition, participating healthcare professionals shared information with family members about palliative care, symptoms and the disease.

*FGP2*: ‘We tell them about the disease and possible future symptoms and what to do and whom to contact’.

*FGN4*: ‘We always meet [the family members], or if they don't come to the ward, we call; we get in touch somehow’.

The shared information was strongly dependent on a patient's condition. Healthcare professionals reported informing family members regularly about daily conditions and symptoms, and changes in medication, deterioration or even the death of a patient.

*FGPN1*: ‘Some family members want to know if there have been any symptoms, whether the patient has slept and eaten, whether they have been awake, and whether they have been well’.

*FGPN2*: ‘We call very quickly [to the family members] if there are any changes in a condition [of the patient]’.

The category *requirements for information sharing* describes the objectives and conditions that must be considered when information is shared in the subcategories of *helping a family member to understand a patient's situation*, *providing direct information* and *respecting a patient's will to deny information sharing with family*. The participating healthcare professionals, particularly nurses and practical nurses, perceived *helping a family member to understand a patient's situation* as an important consideration when sharing information with a family member. The healthcare professionals supported family members to understand that the situation was permanent, that the patient's condition was not getting better, and that death was approaching in some cases.

*FGN1*: ‘You can then touch the patient gently. Here, they are sleeping; they look calm, our medication is working, and they don't need to suffer anymore’.

*FGN3*: ‘We repeat that the situation seems bad and that it is not known if the patient will see the next morning’.

The healthcare professionals also reported needing to justify treatments that were carried out in patient care by sharing details about different care procedures, for example, explaining the decisions made about nutrition and medication. Participants felt that family members quite often questioned decisions and procedures, doubted practices and were well‐informed about patient care, which required time and effort from the healthcare professionals to clarify what they were doing and why.

*FGN6*: ‘Family members want to know more about everything nowadays. We have to know how to explain everything and justify those things more than before’.

*FGN3*: ‘Or we need to be able to justify why an unconscious patient is not given a nasogastric tube and nutrition’.

All the information sharing in palliative care situations requires *providing direct information* to family members, as physicians emphasised in particular. Participants reported that they must be straightforward and express themselves with concise, honest language, even if they perceived that the family member did not want to know all of the necessary information. Straightforward language is seen as necessary to help family members understand the patient's condition. Healthcare professionals reported that it was necessary to speak honestly about the severity of the patient's situation.

*FGP2*: ‘It is important to be honest with patients and family members and use the word “death” as well, to use the right terms, to not use a figure of speech or metaphor that someone does not understand’.

*FGP1*: ‘Sometimes, you are forced to directly say things to family members, even though they may not want to hear it’.

Healthcare professionals also reported facing situations in which they must *respect a patient's will to deny information sharing with family*. If the patient does not want information about their condition and care to be shared with family members, healthcare professionals do not have any other option than to act as required to protect their rights, even if a family member insists on being informed. The patient's right to reject information sharing causes healthcare professionals to feel uncomfortable when family members request information, but the patient does not give permission. In these situations, the healthcare professionals experienced that the family member said that the professionals did not give the support and information they needed and acted against the family.

*FGN3*: ‘We must then respect that the patient does not want information shared with family members’.

*FGPN3*: ‘If the patient says that they do not want anyone to be given information, I have to agree. No matter how angry that family member is, I must respect that patient's right to self‐determination’.

#### 5.1.2 Comprehensive support for family members

The second main category consisted of three subcategories: *Supporting family needs*, *facilitating and mediating family members' and patients' togetherness and supportive encounters*.

*Supporting family needs* describes the support that equips family members to cope and consists of the subcategories *taking care of family members' well‐being* and *organising additional support for family members*. *Taking care of family members' well‐being* includes, for example, healthcare professionals asking family members whether they are taking care of their physical health, eating, sleeping and taking their medications. In addition, participants report make sure that family members get enough rest and sometimes encourage them to go home for a period of rest and relaxation.

*FGN8*: ‘I often say, “try to take care of yourself, get some rest, go outside for a while”, or sometimes just “have you remembered to take your own medicine?”’.

*FGP1*: ‘You also need to evaluate how the family member is doing’.

In addition, the healthcare professionals take family members' wishes into account. Family members' desire to bring a favourite dish or even pets to the care unit, or to engage in religious or cultural practices, are respected.

*FGP1*: ‘The wishes have been recorded by the palliative care coordinator, and the wishes of the family members have been taken into account’.

*FGPN1*: ‘And there are people who need a scarf on their head if a male nurse comes in, so we need to anticipate that’.

Healthcare professionals support family members by *organising additional support for them*. This support may include making appointments with other professionals that can help family members through their crises, such as psychologists, hospital pastors or crisis centres.

*FGN2*: ‘If there is an acute case where the patient has just died, we will contact the crisis services’.

*FGP2*: ‘We also ask if the family member wants a conversation with the hospital pastor’.

The category of *facilitating and mediating family members' and patients' togetherness* includes matters that make family members' involvement in the patient's life easier in the hospital environment. *Creating confidence that the patient receives good care* is a supportive element that participants described as meaningful to family members. Creating an atmosphere of safety and care around the patient gains family members' trust, confidence and perception of being supported. The healthcare professionals in this study perceive that this confidence helps family members release their responsibilities and spend time independently. Confidence and trust in good patient care is understood to enhance family members' coping abilities, allowing them to relax and take breaks from attending to the patient care.

*FGPN3*: ‘And creating a safe, confident feeling for family members so that they could leave here and have trust’.

*FGP1*: ‘The feeling of security conveys that the patient is well cared for’.

Additionally, healthcare professionals report *enabling the presence of family members* so that family members can visit the care unit outside of visiting hours; they can stay with the patient overnight and are free to come and go at will. Participants also try to arrange a dedicated space for family members and private rooms for their patients.

*FGN7*: ‘They can always come here; they can always be here and are welcome’.

*FGP1*: ‘The aim is to have a single room if a family member wants to stay’.

*Encouraging family members' participation* means they are encouraged to come to the care unit, spend time with the patient and participate in the care procedures despite the patient's condition. In addition, healthcare professionals try to promote family members' presence because they know that these could be the last opportunities for family members to spend time with the patient.

*FGN1*: ‘We make the family members feel useful because basic care is what they can participate in. They can help with the feeding and bathing and take care of those things’.

*FGP1*: ‘I usually encourage family members to come to the care unit if they hesitate; their presence cannot be replaced later’.

*Supportive encounters* are implemented by being present, having discussions and listening to the family members. *Healthcare professionals' compassionate presence with family members* is described as warm, understanding encounters with the family members, in which providers gently take into account the uniqueness of the situation, giving a hug and speaking in a soothing tone to create a calm atmosphere.

*FGN3*: ‘They should be treated more gently because the situation is always unique … remember that it is a unique situation for that family’.

*FGPN1*: ‘And I can say, “I can stay here with you for a little while. There's nothing to fear”’.

*Conversations with family members* was experienced as a vital element of support. Healthcare professionals reported speaking with family members about their needs and wishes. They respect the variety of family circumstances and perceive that not all family members want to have conversations with them. Further, participants observed that conversations with family members are recurrent and require a great deal of time. In fact, discussions with family members are perceived to occur even more frequently than discussions with patients.

*FGP2*: ‘We to talk with family members even more often than with the patient’.

*FGN4*: ‘Some families are very open; they want to discuss everything. … Some withdraw; they don't want to talk … and we should be able to respond to it’.

During these discussions, healthcare professionals support family members by listening to their stories about the patient, their lives, the family members' situations and the patient's illness, asking questions to help the family members express themselves.

*FGN5*: ‘Also listen to family members; they want to tell stories about the patient or their situation and going through the illness’.

*FGPN3*: ‘We support them by listening to those stories and guiding in these matters’.

#### 5.1.3 Methods to improve support for family members

In the third main category, perceptions of improving the support for family members included three subcategories: *allocating resources to family members' support*, *coordinating formal and informal support for family members and developing personnel's competence*.

*Allocating resources to the family members' support* means that the healthcare professionals feel they should be given more resources from the organisation to support families. *A better physical environment for families* includes the need for improved facilities to enable family members to remain at the care unit and be present with the patient. Additionally, healthcare professionals desire a private space to have conversations with family members, as under the current circumstances, they find it difficult to avoid breaching confidentiality when there is no dedicated space for these conversations.

*FGN6*: ‘There should be a space, a room for the families’.

*FGPN3*: ‘Well, I wish that we could have a space where you could discuss with a family member because it is not allowed to have conversations at the doors of the rooms’.

Participants also want *more time for encounters with families* because current timelines and resources in the care units do not allow for adequate support or time to meet and talk with the families, even if the providers want to do so.

*FGN7*: ‘They look at the number of patients and not how much time we spend talking to family members. It is not a concrete task and cannot be scheduled, and there should be more time for it’.

*FGP2*: ‘If Thursday's schedule has three family meetings and ten new patients, then there is not enough time [to take the family members into account]’.

*Coordinating formal and informal support for family members* describes support activities that should be dedicated to the family members. In general, the healthcare professionals described a need for *better consideration of family support*, expressing the hope that family members could be taken into account earlier, more often, and more systematically.

*FGP2*: ‘Perhaps a certain systematicity and structure in support would make it available to everyone’.

*FGN3*: ‘That family members could be involved at an earlier stage … that they could hear those things in advance’.

*The development of bereavement support* was another aspect that healthcare professionals perceived as needing improvement. Again, the bereavement support was considered necessary to the family members but has not yet been systematically implemented.

*FGN2*: ‘What I would like to see developed would be the bereavement support for family members’.

*FGP2*: ‘Bereavement support, taking care of family members after a patient's death, a monitoring system should be developed’.

*Informal events for families* were mentioned as a development need because family members desire the opportunity to network with other families and meet with healthcare professionals in informal contexts.

*FGPN2*: ‘Cooperation with family members should be mapped out, or if they want to network and talk to each other, such events should be organised for them’.

*FGP2*: ‘Family members' evenings, or something like that, could be used to meet the healthcare staff too, and maybe family members could find support from each other’.

*Developing professional competence* was described as a necessity. Participants reported that they would benefit from improved communication skills and the ability to speak straightforwardly and have better encounters with the family members.

*FGPN2*: ‘There should be some training for us [about dealing with family members]; interacting here is important, and encountering the families is highlighted’.

*FGP2*: ‘So, I think the healthcare professionals could develop their communication’.

The healthcare professionals in this study also said a desire for *education on family care*. Training was a recurring theme that was suggested as a way to improve the support for family members. Healthcare providers feel that they are professional in their patient care, but their knowledge of family care needs further development.

*FGPN1*: ‘It would be nice to have some training’.

*FGP1*: ‘Education and experience are probably the best teachers [to improve support]’.

## 6 DISCUSSION

This study presented how psychosocial family support was implemented in hospital palliative care units from the perspective of healthcare professionals involved in the daily care of palliative care patients. The healthcare professionals in this study perceive that the psychosocial support they provide includes informational and comprehensive support for family members, and they also made suggestions about how they experience that support could be improved in their care practices.

Information sharing is recognised as an essential element of support, and the healthcare professionals experience that they carried out communication actively, whether the family members were at the care unit or home. The importance of fair and honest information sharing, communication and the effort to make family members understand the palliative care situation has been observed to promote family support (Anderson et al., 2019; Røen et al., 2019), and the healthcare professional's role implementing informational support is essential to the families as they are the ones whose family members encounter in hospitals, can ask questions and have conversations with about their concerns. However, the healthcare professionals experience as a negative factor that the level of knowledge and information‐seeking abilities of family members has developed and increased. Family members' opinions and questions about healthcare professionals' decisions are not without problems, and the need to constantly justify the treatments, medications and patient care to family members is perceived as excessively demanding by the healthcare professionals. Nevertheless, family members are an essential part of patient care, and their need for information and desire to participate is reasonable, and if the healthcare professionals' attitudes towards the family members are negative, there is a risk that it hinders the realisation of information sharing and support (Partanen et al., 2018). It could be beneficial to enhance family‐centredness in palliative care environments, so the family members' questions and information seeking would be seen as a positive factor to cooperate for the benefit of the patient's care.

Participants described making many efforts to provide support to promote family members' coping by taking care of their well‐being and resilience. Particularly, the importance of practising empathy, having conversations and listening were highlighted. A patient's palliative care situation was seen as a unique event for family members, and participants recognise that they must be treated with particular care. However, the healthcare professionals experienced that the recurrent conversations with family members were demanding and time‐consuming, which might indicate the family members' burden and anxiety during palliative inpatient care, emphasising the need for support. In addition, the healthcare professionals described spending considerable resources supporting family members' coping. The healthcare professionals attended to family members various needs and wishes, made efforts to create confidential care relationships and enabled family members' presence and participation as much as possible. However, even though healthcare professionals experience they had make efforts and spend many resources to support the families, the perspective of family members often reveals that their informational or emotional support is not sufficient or that their care needs are not met enough (Røen et al., 2019; Saarinen et al., 2021; Soikkeli‐Jalonen et al., 2021; Virdun et al., 2017). According to these results, there must be some discrepancy between the healthcare professional's perception of support provided to families and its perceived benefits compared to family to respond to them.

Furthermore, the importance of family members' own experiences, indicating that although support was given to families, the current support may not always be efficient. More attention should be aimed at recognising family members' need for support and healthcare professionals' opportunities to respond to them.

Furthermore, the importance of family members' participation in patient care was noticed and encouraged by the healthcare professionals. In cancer care settings, family members' closeness to the patient, the care environment, culture and attitudes in the care unit all play vital roles in supporting the family members (Partanen et al., 2018). Healthcare professionals specialising in palliative care saw similar aspects of support as essential. Healthcare professionals said that promoting patient and family togetherness by encouraging the family members' presence and participation is an important supportive element. Furthermore, they perceive that making the environment respective and the atmosphere welcoming makes the family feel supported. In addition, confidential relationships and family members' trust in good patient care are believed to enhance family members' coping abilities. As the importance of acceptance, feeling welcome and promoting family participation are acknowledged by both, the families and healthcare professionals, these important aspects should be better implemented in daily care. At the moment, healthcare environments do not consider families and their needs enough (Røen et al., 2019; Salifu et al., 2021) and family members feel often as outsiders in hospital care (Ullrich et al., 2021).

The healthcare professionals in this study made suggestions for improving practices to support family members, including resources, the need for systematic support and strengthening their competencies in family care. The healthcare professionals said that their organisations had not allocated sufficient resources to the support of families. Patient care is seen as the priority, and the healthcare professionals do not have enough time to meet and talk with the families, although they want to. However, family members should always be an integral part of palliative care and must be cared for and patients (Steele & Davies, 2015). The need for the comprehensive integration of families still requires more attention when allocating healthcare resources in the palliative care context. The healthcare professionals said a need for better facilities for family members to have their own space in the care unit, the ability to stay overnight and a space to have conversations in private. In general, support focusing exclusively on family members is needed. A systematic support system for family members is perceived to be necessary because currently there is no form of help or guidance for how healthcare providers should assist family members. The healthcare system was constructed to focus on patients, and systematic support options are needed. The findings of this study align with the observation that support opportunities, particularly interventions directed at family members in inpatient palliative care, rarely exist (Saarinen et al., 2021; Soikkeli‐Jalonen et al., 2021). Furthermore, an organised bereavement support system is lacking, even though bereavement support would be helpful to assist family members in coping with the grieving process (Kustanti et al., 2021). In addition, the healthcare professionals in this study express that they would benefit from receiving training in family care in a palliative care setting, and this lack of sufficient training in family care has been observed in a previous study as well (Teixeira et al., 2019).

### 6.1 Strengths and limitations

Purposive sampling may have influenced the selection of participants, but it facilitated the discovery of eligible informants with experience relevant to the topic. The participants included physicians and nursing staff, so the perspectives of healthcare professionals who do not participate in the regular care of patients and families were not included. The study participants included more nurses and practical nurses than physicians, which may have given greater emphasis to their perspective. However, nursing staff represent the largest professional group in inpatient units, and the number of participants is proportional to the number of different professions in the units, as generally, there is only one physician per ward. The interviews were carefully planned and constructed such that all the interviews were conducted as similarly as possible. The data collection, performed as a whole without separating perspectives of professions, reached a point of saturation, increasing the credibility of the results. As a descriptive study, the code saturation was perceived as sufficient, and the meaning saturation was not considered necessary (Hennink et al., 2017). However, data saturation about different professional groups is not certain. The data analysis was performed in a research group, with the following consolidated criteria for reporting qualitative studies (COREQ): 32‐item checklist was used to ensure detailed and comprehensive reporting during the analysis and reporting processes (Tong et al., 2007). The participants were unable to see the transcripts or results afterwards to add comments, corrections or feedback, because the interviews were conducted in groups and lasted rather long (see Table 1), and in written they comprised long transcripts, so the healthcare professionals time resources would not allow them to delve into them sufficiently. Additionally, identifying and recognising paragraphs that particular persons had said was very difficult from the written transcripts, and the participants would not get enough information about them.

## 7 CONCLUSIONS

The healthcare professionals describe their perceptions of psychosocial support for family members primarily through patient care and the patient's situation rather than family needs. Direct support for the family members is realised principally by information sharing and conversations, but the other ways of supporting families were not explained much. Healthcare professionals express their opportunities to implement support focusing on family members' needs restricted; organisational resources, such as the time needed to meet the families and appropriate space and environments for family members, were limited in inpatient units. The facilities were not always suitable to support family members' presence and participation. Additionally, there was a need for coordinated and organised support systems for family members that could be implemented in inpatient units by the healthcare professionals as a part of the daily care. Systematic support programmes or models designed for family members and organised bereavement support are needed. Furthermore, additional training for healthcare professionals in palliative family care is needed.

## AUTHOR CONTRIBUTIONS

Anu Soikkeli‐Jalonen: Conceptualisation, methodology, investigation, data curation, writing (original draft), reviewing, editing, visualisation and funding acquisition. Kaisa Mishina: Methodology, investigation, writing, reviewing, editing and supervision. Heli Virtanen: Investigation, writing, reviewing and editing. Andreas Charalambous: Writing, reviewing, and editing. Elina Haavisto: Conceptualisation, methodology, investigation, data curation, writing, reviewing, editing, funding acquisition, supervision and project administration.

## CONFLICT OF INTEREST

The authors declare that there is no conflict of interest.

## Data Availability

The data that support the findings of this study are partly available upon request from the corresponding author. The data are not publicly available due to privacy or ethical restrictions.
